# Vitamin C Intake is Inversely Associated with Cardiovascular Mortality in a Cohort of Spanish Graduates: The SUN Project

**DOI:** 10.3390/nu9090954

**Published:** 2017-08-29

**Authors:** Nerea Martín-Calvo, Miguel Ángel Martínez-González

**Affiliations:** 1 Department of Preventive Medicine and Public Health, University of Navarra, 31008 Pamplona, Navarra, Spain; nmartincalvo@unav.es; 2 IdiSNA, Navarra Institute for Health Research, 31008 Pamplona, Navarra, Spain; 3 CIBER Physiopathology of Obesity and Nutrition (CIBERobn), Carlos III Institute of Health, 28029 Madrid, Spain; 4 Department of Nutrition, Harvard T.H. Chan School of Public Health, Boston, MA 02115, USA

**Keywords:** vitamin C, cardiovascular disease, cardiovascular mortality, fiber

## Abstract

Observational studies have found a protective effect of vitamin C on cardiovascular health. However, results are inconsistent, and residual confounding by fiber might be present. The aim of this study was to assess the association of vitamin C with the incidence of cardiovascular disease (CVD) and cardiovascular mortality (CVM) while accounting for fiber intake and adherence to the Mediterranean dietary pattern. We followed up 13,421 participants in the Seguimiento Universidad de Navarra (University of Navarra follow-up) (SUN) cohort for a mean time of 11 years. Information was collected at baseline and every two years through mailed questionnaires. Diet was assessed with a validated semi-quantitative food frequency questionnaire. Incident CVD was defined as incident fatal or non-fatal myocardial infarction, fatal or non-fatal stroke, or death due to any cardiovascular cause. CVM was defined as death due to cardiovascular causes. Events were confirmed by physicians in the study team after revision of medical records. Cox proportional hazard models were fitted to assess the associations of (a) energy-adjusted and (b) fiber-adjusted vitamin C intake with CVD and CVM. We found energy-adjusted vitamin C was inversely associated with CVD and CVM after adjusting for several confounding factors, including fiber from foods other than fruits and vegetables, and adherence to the Mediterranean dietary pattern. On the other hand, when vitamin C was adjusted for total fiber intake using the residuals method, we found a significant inverse association with CVM (HR (95% confidence interval (CI)) for the third tertile compared to the first tertile, 0.30 (0.12–0.72), but not with CVD in the fully adjusted model.

## 1. Introduction

Vitamin C, also known as L-ascorbic acid, is a water-soluble vitamin naturally present in some foods, added to others, and available as dietary supplement. Vitamin C is an essential dietary component, since humans, unlike most animals, are unable to synthetize it. Vitamin C is required for the synthesis of collagen, L-carnitine and some neurotransmitters. Based on vitamin C’s antioxidant capacity, there is growing interest in assessing whether vitamin C intake might help prevent or delay some type of cancer, cardiovascular disease (CVD) or other diseases in which oxidative stress plays an important role.

Recommended dietary allowances (RDA) for vitamin C—75 mg/day for women and 90 mg/day for men [1]—are based on its known physiological and antioxidant functions in white blood cells and are higher than the amount required to prevent deficiency. Nevertheless, given that vitamin C may relate to cancer, CVD, or other diseases through different mechanisms, whether classical RDAs are optimal to obtain maximum benefits is unclear [2].

The belief that vitamin C relates to cardiovascular health stemmed from the benefits observed from fruit and vegetable consumption [3,4,5]. Observational studies have found an inverse association of dietary vitamin C [6] and ascorbic acid plasma levels [6,7,8] with cardiovascular risk factors, CVD, and cardiovascular mortality (CVM). Nevertheless, those studies showed some limitations, including suboptimal adjustment for potential confounders such as fiber intake.

Vitamin C from foods and supplements seemed to be equally bioavailable [9]. However, observational studies [6] and clinical trials [10,11,12,13] concluded that supplementation with vitamin C (500 to 1000 mg/day) had no effect on different cardiovascular endpoints. Moreover, higher CVM and total mortality has been reported among participants under vitamin C supplementation in both observational [14] and interventional studies [15]. On the other hand, two meta-analyses reported that high dose supplementation with vitamin C ((500 to 2000 mg/day) and (500 to 4000 mg/day) respectively) was associated to endothelial function improvements [16] and reduced blood pressure [17].

The aim of this study was to assess whether vitamin C intake was independently associated with lower CVD and CVM risk among participants in the Seguimiento Universidad de Navarra (University of Navarra follow-up) (SUN) cohort.

## 2. Materials and Methods 

### 2.1. Study Population

The SUN project is an ongoing, prospective and multipurpose cohort of Spanish university graduates. As a dynamic cohort, enrolment is permanently open, and follow-up information is gathered by mailed questionnaires every two years. Regarding the obtention of informed consent of potential participants, we duly informed these potential candidates of their right to refuse to participate in the SUN study or to withdraw their consent to participate at any time without reprisal, according to the principles of the Declaration of Helsinki. Special attention was given to the specific information needs of individual potential candidates as well as to the methods used to deliver their information and the feedback that may receive in the future from the research team. After ensuring that the candidate had understood the information, we sought their potential freely-given informed consent, and their voluntary completion of the baseline questionnaire. These methods were accepted by our Institutional Review Board as to imply an appropriately-obtained informed consent. A more detailed description of the SUN methodology can be found elsewhere [18]. The study protocol was approved by the Institutional Review Board of the University of Navarra (approval code 010830).

**Figure 1:**
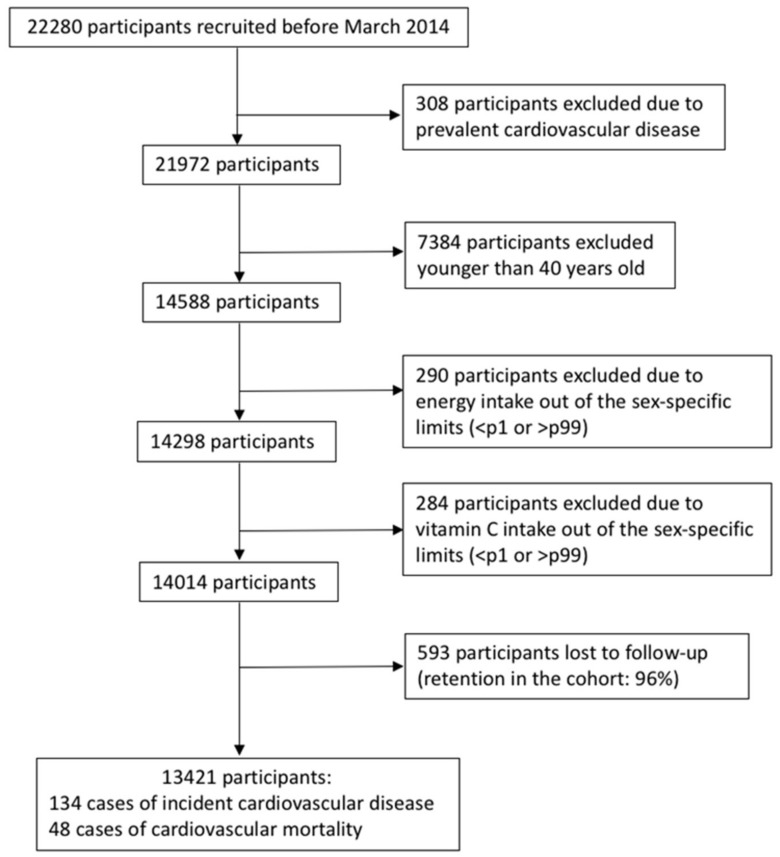
Flow chart of participants for the assessment of the association of cardiovascular disease and cardiovascular mortality with vitamin C intake in the Seguimiento Universidad de Navarra (University of Navarra follow-up) (SUN) cohort (follow-up 1999–2016).

We assessed 22,280 participants recruited before March 2014 to ensure they completed at least the two-year follow-up questionnaire. We excluded 308 participants due to prevalent cardiovascular disease, 7384 participants younger than 40 years old who were considered too young to present a cardiovascular event during the follow-up, 290 participants with energy intake out of the sex-specific limits (under p1 or above p99), and 284 participants with vitamin C intake out of the sex-specific limits (under p1 or above p99). Out of the rest of the participants, 593 were lost to follow-up (retention in the cohort: 96%), leading to a final sample of 13,421 participants (Figure 1).

### 2.2. Exposure Assessment

Participants were asked to complete a previously validated semi-quantitative food frequency questionnaire (FFQ) [19,20] and report how often on average they had consumed 136 foods and beverages during the past year. The FFQ had nine categories for intake frequency, from never to two or more servings per day. Multivitamin and supplements users were asked to specify the brand of multivitamin or supplement, the dose, and frequency of use. The nutritional content of each food was obtained from Spanish food composition guides [21,22] and supplemented with information from food and supplement manufacturers when needed. The nutrient contribution of each food item was calculated by multiplying the frequency of food consumption by the nutrient composition of the specified portion size. Dietary vitamin C intake was adjusted for energy intake using the residuals method, and categorized into tertiles. Total vitamin C intake was estimated by summing the vitamin C contribution of food items and supplements. In ancillary analyses, we assessed the independent effect of vitamin C from foods additionally adjusted for supplement intake (dichotomous variable).

In further analyses, to nullify the correlation between vitamin C and fiber, dietary vitamin C was alternatively adjusted for total fiber intake using the residuals method.

### 2.3. Outcome Assessment

Incident CVD was defined as either incident fatal or non-fatal myocardial infarction (with or without ST elevation), or fatal or non-fatal stroke and death due to other cardiovascular causes. CVM was defined as death due to cardiovascular causes.

Information about the events was initially gathered from follow-up questionnaires. When participants reported any of the previously mentioned events, they were asked for their medical reports, which were evaluated by physicians in the study team who were blinded to the nutritional information. Myocardial infarction was diagnosed using universal criteria. Non-fatal stroke was defined as sudden onset focal-neurological lack with a vascular mechanism that last more than 24 h. Confirmed events were classified according to the International Classification of Diseases (ICD-10). I21 and I63 codes were considered to define cardiovascular events [23]. The National Death Index is checked at least once a year to confirm the vital status of participants during follow-up. Deaths were reported by either participant’s next of kin, work associates, or postal authorities.

Participants were followed-up from enrollment until December 2016, the diagnosis of the event, or death, whichever came first.

### 2.4. Covariates

Information about socio-demographic and anthropometric characteristics, lifestyle (physical activity, television watching, smoking status), classical cardiovascular risk factors (hypertension, hypercholesterolemia, hypertriglyceridemia and diabetes), prevalent diseases (cancer and cardiovascular related diseases), and family history of stroke and cardiovascular-related medication was collected at baseline.

Age was calculated as the difference between the date of recruitment and the date of birth. Body mass index (BMI) was calculated by dividing participants’ weight (kg) by their squared height (m). A validation study in a subset of the SUN cohort showed that self-reported weight and height were highly reproducible and specific [24].

Dietary information was obtained from the baseline FFQ. Energy (kcal/day) and fiber (mg/day) intakes were calculated by multiplying the frequency of each food item consumed by the energy and fiber contribution of its specified portion size. Total energy and fiber intakes were calculated as the sum of energy and fiber provided by each food item. We also calculated the adherence to the Mediterranean dietary pattern based on the information from the FFQ using the classical Mediterranean Dietary Score (MDS) [25] without the fruit- and vegetable-related items (total seven items). We defined three categories of adherence to the MDS: Low (from 0 to 2 points), medium (from 3 to 4 points), and high (from 5 to 7 points).

Physical activity was collected at baseline with a previously validated questionnaire [26] that included 17 activities and 10 categories of response, from never to eleven or more hours per week. METs-h/week for each activity were calculated by multiplying the number of Metabolic Equivalent of Task (METs) of each activity [27] by the weekly participation in that activity, weighted according to the number of months dedicated to each activity. Total physical activity was quantified by summing the METs-h/week dedicated to all activities performed during leisure time. Time spent watching television was used as a proxy of sedentary behavior [28]. Hours per week of television watching were calculated as the mean of hours spent watching television during weekdays and hours spent watching television during weekends. Missing data were imputed based on the values of other covariates.

Cardiovascular-related diseases at baseline (coronary heart disease, tachycardia, atrial fibrillation, aortic aneurism, heart failure, venous thrombosis, and claudication) were grouped in a single quantitative variable (number of cardiovascular-related diseases) included in the multivariable adjustment. Validation studies in the SUN cohort showed self-reported information about cardiovascular risk factors was valid as to be used in epidemiological studies [29,30].

### 2.5. Statistical Analysis

Baseline characteristics of participants were presented by tertiles of total vitamin C intake as mean (standard deviation) for quantitative variables, and as proportions for qualitative variables. A *p* value for trend across tertiles was calculated using simple linear or logistic regressions.

We fitted Cox proportional hazard models to assess the association of energy-adjusted vitamin C intake with CVD and CVM. We estimated the hazard ratios (HR) and their 95% confidence intervals (CI) for second and third tertile of vitamin C intake compare to the lowest tertile (category of reference). Age was used as the underlying time variable in all the models. We fitted five multivariable adjusted models: (1) adjusted for age and sex; (2) additionally adjusted for body mass index (continuous), total energy intake (continuous), physical activity (continuous), television watching (continuous), smoking (never, current, or former), family history of stroke (dichotomus) and treatment with aspirin (dichotomus); (3) additionally adjusted for number of cardiovascular-related diseases at baseline (discrete), prevalent cancer (dichotomus), prevalent hypertension (dichotomus), prevalent diabetes (dichotomus), prevalent hypercholesterolemia (dichotomus) and prevalent hypertriglyceridemia (dichotomus); (4) additionally adjusted for dietary fiber (fiber from foods other than fruits and vegetables) (continuous); and (5) additionally adjusted for adherence to the MDS (without the fruit- and vegetable-related items (low, medium or high).

Interactions with vitamin C supplements intake, total fiber intake and age at the end of follow-up were assessed for both CVD and CVM by adding an interaction product term to the model and calculating the maximum likelihood ratio test.

In ancillary analyses, we evaluated the association of tertiles of dietary vitamin C with CVD and CVM fitting a model additionally adjusted for vitamin C supplements intake (dichotomus).

In further analyses, we re-ran the multivariable adjusted models for fiber-adjusted vitamin C intake categorized into tertiles.

Analyses were performed with STATA version 12.0 (StataCorp, College Station, TX, USA).

## 3. Results

We followed-up 13,421 participants for a mean time of 10.9 years (the standard deviation (SD) = 3.82). Baseline characteristics of participants by tertiles of vitamin C intake are described in Table 1. Participants in the highest tertile of vitamin C intake (from 320 to 1110 mg/day) were older, more likely to be women, and less likely to be current smokers. They were also more physically active and spent less time watching television. Moreover, they reported higher fiber intake and greater adherence to the Mediterranean dietary patter (MDP). We found total vitamin C intake showed a modest correlation with energy intake (*r* = 0.33), but it was highly correlated with total fiber intake (*r* = 0.72). Similar results were found for dietary vitamin C.

Aortic aneurism, heart failure, and hypertriglyceridemia at baseline were less prevalent among participants with higher vitamin C intake. However, cancer, venous thrombosis, diabetes, hypertension, and family history of stroke at baseline were more prevalent, probably due to the older age of participants with higher intake of vitamin C. Participants in the highest tertile of vitamin C intake were more likely to be under treatment with diuretics, antihypertensives, aspirin, and other cardiovascular treatment drugs.

**Figure 2:**
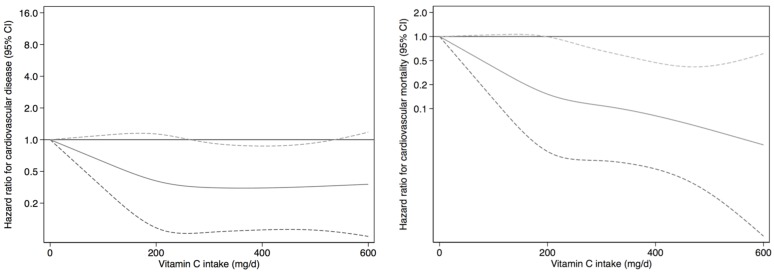
Restricted Cubic Splines for the Hazard Ratio (HR) and 95% Confidence Interval (CI) for cardiovascular disease and cardiovascular mortality associated with total vitamin C intake in the SUN cohort (follow-up 1999–2016). Age strata as underlying time variable. Multivariable model adjusted for sex, body mass index (continuous), total energy intake (continuous), physical activity (continuous), television watching (continuous), smoking (never, current or former), family history of stroke, treatment with aspirin, number of cardiovascular-related diseases at baseline, prevalent cancer, prevalent hypertension, prevalent diabetes, prevalent hypercholesterolemia, prevalent hypertriglyceridemia, fiber (from foods other than fruits and vegetables) (continuous), and Mediterranean Dietary Score (MDS) without fruit- and vegetable-related items (low, medium, high).

Multivariable-adjusted associations of total vitamin C intake with both CVD and CVM are showed in Figure 2.

### 3.1. Cardiovascular Disease

A total of 134 cases of CVD were identified over 146,973 person-years at risk. The cumulative risk of a cardiovascular event was 0.07% in the highest tertile versus 0.12% in the lowest tertile of vitamin C intake.

We found that higher vitamin C intake was associated with a lower risk of CVD in the age-adjusted analysis (Table 2). Moreover, this association remained significant in the age and sex-adjusted model, in the model adjusted for demographic, metabolic, and lifestyle risk factors (multivariable adjusted model 1), and in the model additionally adjusted for prevalent diseases at baseline (multivariable adjusted model 2). Further adjustment for fiber intake (multivariable adjusted model 3) did not change the results. In the fully adjusted model (multivariable adjusted model 4), we found that, compared with participants in the first tertile of vitamin C intake, those in the second and third tertiles showed significant lower risk of CVD (HR (95% CI): 0.60 (0.40–0.91) and 0.62 (0.40–0.97), respectively).

High vitamin C intake showed no significant association with CVD when fiber from fruits and vegetables was also considered. HRs (95% CI) for the third tertile in models 3 and 4 were 0.66 (0.39–1.10) and 0.68 (0.40–1.13), respectively. Nevertheless, the association was still significant when the second and third tertiles were considered together (HR (95% CI): 0.63 (0.41–0.94) for model 3 and 0.64 (0.43–0.88) for model 4).

Neither age at the end of follow-up (*p* = 0.79) nor fiber intake (*p* = 0.15) resulted in effect modification. Marginally significant interaction was found between total vitamin C and vitamin C supplementation (*p* = 0.05).

### 3.2. Cardiovascular Mortality

A total of 48 cases of CVM occurred over 147,495 person-years at risk during the follow up. The cumulative risk was 0.02% in the third tertile versus 0.04% in the first tertile of vitamin C intake.

Compare to the category of reference, we found a significant inverse association for the highest tertile of vitamin C intake and CVM in the age-adjusted analysis (Table 3). Results were similar in the age and sex-adjusted model; the models adjusted for demographic, metabolic and lifestyle risk factors; (multivariable adjusted model 1); and in the model additionally adjusted for prevalent diseases at baseline (multivariable adjusted model 2). Additional adjustment for fiber from foods other than fruits and vegetables did not change the results, but they became non-significant when total fiber intake was considered (HR (95% CI): 0.48 (0.19–1.20)). No significant results were found in the fully-adjusted model that included the MDS.

Neither age at the end of follow-up (*p* = 0.70), fiber intake (*p* = 0.42), nor vitamin C supplements intake (*p* = 0.12) modified the association between total vitamin C intake and CVM.

### 3.3. Fiber-Adjusted Vitamin C Intake

In further analyses, dietary vitamin C was adjusted for total fiber intake using the residuals method to nullify the correlation between vitamin C and fiber (Table 4). We found a cumulative risk for CVD of 0.07% in the third versus 0.12% in the first tertile. However, no significant association was found for vitamin C intake and CVD.

On the other hand, the cumulative risk for CVM was 0.01% in the third tertile versus 0.05% in the first one. Compared with participants in the first tertile, those in the highest tertile of vitamin C intake showed significant lower risk of CVM in multivariable adjusted analyses (HR (95% CI): 0.30 (0.13–0.73)). Further adjustment for the MDS did not change the results.

Neither CVD nor CVM were significantly associated with dietary vitamin C, independently of vitamin C supplement intake (Figure S1).

## 4. Discussion

In this large cohort of Spanish university graduates followed-up over a mean time of 11 years, we found that, compared with the lowest category, the third tertile of total vitamin C intake was associated with 70% (95% CI 18%–88%) lower risk of CVM, but not with CVD. This analysis was based on a multivariable adjusted model that thoroughly controlled potential confounding by fiber and accounted for the adherence to the Mediterranean dietary pattern.

The belief that vitamin C benefits cardiovascular health is based on its antioxidant capability. Vitamin C may prevent oxidative changes to low-density lipoprotein (LDL)-cholesterol [31] and reduce monocyte adhesion [32], which are key in reducing the risk of atherosclerosis. Moreover, vitamin C prevents vascular smooth muscle cells apoptosis, which keeps atheroma plaques stables [33]. In addition, vitamin C improves the nitric oxide production of the endothelium [34], which in turn contributes to reduced blood pressure. This evidence, when added to the results found in the analyses to account for confounding by fiber and dietary variables included in the MDS, suggests that the associations of vitamin C intake with CVM may not be due to confounding factors, but may instead represent a true biological effect.

Observational studies had reported inverse associations of vitamin C with cardiovascular outcomes, particularly on hypertension [6] and heart failure [7]. However, those studies did not account for fiber intake. Due to the high correlation between vitamin C and fiber intakes found in this study, it was difficult to assess the effect of vitamin C on cardiovascular health independently of fiber intake in a multivariable adjusted model. In order to nullify that correlation, dietary vitamin C was adjusted for total fiber intake using the residuals method. On the other hand, reduced CVM risk associated to vitamin C intake had been previously reported in observational studies [8]. However, this association has not been confirmed in randomized controlled trials [10,11].

We found that energy-adjusted total vitamin C intake was associated with a lower risk of CVD. We obtained similar estimates in the comparisons of the second and the third tertiles, which suggests a threshold effect or L-shaped association between total vitamin C and CVD. However, in further analyses, we found the association of fiber-adjusted vitamin C with CVD was not significant.

We also found that high energy-adjusted total vitamin C intake was associated with lower risk of CVM after multivariable adjustment for demographic, metabolic, and lifestyle risk factors, prevalent diseases at baseline, and fiber from foods other than fruits and vegetables. However, results became non-significant when the model was additionally adjusted for the MDS. Nevertheless, further analyses showed that, compared to the first tertile, the highest category of fiber-adjusted vitamin C intake was associated with lower CVM risk in the fully adjusted model (HR: 0.30, 95% CI (0.12–0.72)).

These results suggest that most of the confounding effect by fiber was due to fiber from fruits and vegetables. Since vitamin C and fiber were highly correlated (*r* = 0.72) it was difficult to assess the effect of one of them while keeping the other one constant (Table 2 and Table 3). When vitamin C was adjusted for fiber using the residuals method (Table 4), the correlation was nullified (*r* = 0), which allowed for the assessment of the effect of vitamin C on cardiovascular health independently of fiber. Nevertheless, given that vitamin C is a single nutrient and may not represent the whole dietary pattern, these results must be taken with caution. Several reasons support the hypothesis that attributing all of the observed effect to a single nutrient or food may be too simplistic and that when assessing the association of dietary variables with non-communicable diseases, the whole dietary pattern should be considered [35].

None significant associations with either CVD or CVM were found when vitamin C from food was considered alone. Importantly, means (SD) (mg/day) of fiber-adjusted dietary vitamin C intake across successive tertiles were 184 (57), 266.7 (20.5), and 387.7 (75.6) respectively. Therefore, the absence of significant results might be explained by the low variability in the exposure.

Regarding vitamin C supplements, our results parallel previous intervention studies that reported no effect of vitamin C supplementation on cardiovascular health [10,11,12,13]. It must be acknowledged that some clinical trials permitted the control group to an intake of vitamin C and multivitamin supplements, which made it harder to find significant differences between groups. Vitamin C supplementation in our study ranged from 3.4 to 440 mg/day, which is much lower than the doses assessed in the available clinical trials. We found the effect of total vitamin C on CVD may depend on vitamin C supplementation (*p* for interaction 0.05). However, among the 1055 participants undertaking vitamin C supplementation (8%), we found two cases of CVD and one single case of CVM; thus, stratified analyses were not possible.

Some limitations of this study must be acknowledged. First, because information about exposure was self-reported, some degree of misclassification is possible. Nevertheless, information bias would more likely be non-differential with respect to the outcomes, resulting in an attenuation of the observed associations. Moreover, little variability observed in the exposure might have reduced the possibility of significant findings. Second, the SUN cohort is not a representative sample of the general population, and therefore generalization of these results must be based on biological mechanisms rather than on statistical representativeness. Third, given the observational design of the study, the possibility of residual confounding for factors that were not considered (such as vitamin E) must be taken into account. Thus, before causality is implied, these results must be confirmed in well-designed randomized controlled trials. Finally, because participants in the SUN cohort are relatively young and health conscious, few incident cases of CVM were observed during follow-up. Further studies are need to determine if the magnitude of the association we observed represents the upper bound of the association between vitamin C and CVM. Despite these limitations, our study has several strengths. The sample size is large, the follow-up period is long, and the retention rate is high. Dietary information was collected with a validated FFQ, and outcomes were confirmed by physicians checking participant’s medical records. Finally, participants in the SUN cohort are highly educated, and more than half are health professionals themselves, which reduces potential confounding by educational level, leads to better quality in self-reported data, and increases the internal validity of the study.

## 5. Conclusions

Energy-adjusted analyses suggest a threshold effect in the association of vitamin C intake with CVD, but not with CVM. Nevertheless, the model fitted to thoroughly control potential confounding for fiber showed that compared with the category of reference, the highest tertile of total vitamin C intake was associated with a significantly lower risk of CVM, but not CVD, after adjusting for several confounding factors, including adherence to the Mediterranean dietary pattern. Further research is needed in order to fully understand the biological mechanisms explaining these associations. Moreover, these results must be reproduced in different populations before clinical implications can be assessed.

## Figures and Tables

**Table 1:** Baseline characteristics of participants over 40 years old in the SUN cohort by tertiles of total vitamin C intake. Numbers are means (SD) or percentages.

Baseline Characteristics		Tertiles of Vitamin C Intake			
		Q1	Q2	Q3	*p*
*N*		4474	4474	4473	
Vitamin C intake (mg/day)		148 (44.2)	257 (33.0)	445 (114)	<0.001
Fiber intake (g/day)		23.0 (10.0)	27.8 (9.8)	38.3 (14.1)	<0.001
Vittamin C range (mg/day)		0–205	206–319	320–1110	
Vittamin C from supplements (mg/day)		0.56 (4.2)	2.0 (10.0)	9.6 (33.4)	<0.001
Sex (female)		41.6	55.8	67.9	<0.001
Age (years)		41.2 (10.3)	42.8 (10.7)	43.7 (10.8)	<0.001
BMI (kg/m^2^)		24.3 (3.6)	24.1 (3.5)	23.8 (3.5)	<0.001
Mediterranean Dietary Score ^§^					<0.001
	Low (0–2 points)	39.0	29.6	21.4	
	Medium (3–4 points)	47.3	50.9	51.1	
	High (5–7 points)	13.7	19.5	27.5	
Energy intake (kcal/day)		2548 (804)	2346 (710)	2530 (755)	0.26
Physical activity (MET-h/week)		23.4 (20.6)	25.8 (21.6)	29.2 (25.3)	<0.001
Television time (h/week)		1.63 (1.1)	1.57 (1.1)	1.51 (1.1)	<0.001
Family history of myocardial infarction		15.8	18.3	17.1	0.09
Smoking					0.03
	Never	43	44	44	
	Current	29	23	22	
	Former	28	33	34	
Prevalent diseases					
	Cancer	3.7	4.6	5.6	<0.001
	Coronary heart disease	0.38	0.47	0.27	0.39
	Tachycardia	1.9	1.6	2.3	0.12
	Atrial fibrillation	0.65	0.72	0.69	0.80
	Aortic aneurism	0.25	0.11	0.02	0.01
	Heart failure	0.42	0.56	0.38	0.75
	Pulmonary embolism	0.13	0.09	0.11	0.75
	Venous thrombosis	0.51	0.92	0.92	0.03
	Claudication	0.31	0.31	0.56	0.07
	Diabetes	1.4	2.2	3.0	<0.001
	Hypertension	9.7	11.3	10.9	0.07
	Hypercholesterolemia	20.0	21.9	20.7	0.42
	Hypertriglyceridemia	8.5	8.9	7.3	0.04
Drugs					
	Digoxin	0.11	0.13	0.13	0.77
	Diuretics	1.0	1.6	1.7	0.01
	Beta blockers	1.7	2.3	1.9	0.40
	Calcium antagonists	0.40	0.45	0.63	0.13
	Nitrite	0.13	0.11	0.18	0.57
	Antihypertensives	2.8	4.1	3.7	0.03
	Aspirin	3.4	5.2	4.9	0.001
	Other CV treatment drug	5.2	6.9	6.6	0.01

^§^ Mediterranean Diet Score without the fruit- and vegetable-related items. *N* = 13,421.

**Table 2:** Hazard Ratio (HR) and 95% Confidence Interval (CI) for cardiovascular disease (CVD) associated with total vitamin C intake for participants over 40 years old in the SUN cohort (follow-up 1999–2016).

Main Analyses ^§^	Tertiles of Vitamin C Intake		
	Q1 (*N* = 4474)	Q2 (*N* = 4474)	Q3 (*N* = 4473)
Incident CVD (person-years at risk)	61 (50,792)	38 (48,765)	35 (47,415)
Age-adjusted	1.00 (Ref.)	0.52 (0.35–0.78)	0.44 (0.29–0.67)
Sex- and age-adjusted	1.00 (Ref.)	0.59 (0.39–0.89)	0.56 (0.37–0.86)
Multivariable adjusted model 1	1.00 (Ref.)	0.59 (0.39–0.90)	0.60 (0.39–0.93)
T2 + T3 vs. T1	1.00 (Ref.)	0.60 (0.42–0.85)	
Multivariable adjusted model 2	1.00 (Ref.)	0.58 (0.38–0.88)	0.58 (0.37–0.90)
T2 + T3 vs. T1	1.00 (Ref.)	0.58 (0.41–0.83)	
Multivariable adjusted model 3	1.00 (Ref.)	0.58 (0.38–0.88)	0.58 (0.37–0.90)
T2 + T3 vs. T1	1.00 (Ref.)	0.58 (0.41–0.83)	
Multivariable adjusted model 4	1.00 (Ref.)	0.60 (0.40–0.91)	0.62 (0.40–0.97)
T2 + T3 vs. T1	1.00 (Ref.)	0.61 (0.43–0.88)	

^§^ Age strata as underlying time variable in all the models; *N* = 13,421; Ref: reference category. Multivariable adjusted **model 1**: Additionally adjusted for sex, body mass index (continuous), total energy intake (continuous), physical activity (continuous), television watching (continuous), smoking (never, current or former), family history of stroke, and treatment with aspirin. Multivariable adjusted **model 2**: Additionally adjusted for the number of cardiovascular-related diseases at baseline, prevalent cancer, prevalent hypertension, prevalent diabetes, prevalent hypercholesterolemia and prevalent hypertrygliceridemia. Multivariable adjusted **model 3**: Additionally adjusted for dietary fiber (fiber from foods other than fruits and vegetables) (continuous). Multivariable adjusted **model 4**: Additionally adjusted for the MDS without fruit and vegetable intake related items (low, medium, or high).

**Table 3:** Hazard Ratios (HR) and 95% Confidence Intervals (CI) for cardiovascular mortality associated with total vitamin C intake for participants over 40 years old in the SUN cohort (follow-up 1999–2016).

Main Analyses ^§^	Tertiles of Vitamin C Intake		
	Q1 (*N* = 4474)	Q2 (*N* = 4474)	Q3 (*N* = 4473)
Cardiovascular deaths (person-years at risk)	22 (51,016)	15 (48,901)	11 (47,577)
Age-adjusted	1.00 (Ref.)	0.55 (0.28–1.06)	0.34 (0.17–0.73)
Sex- and age-adjusted	1.00 (Ref.)	0.56 (0.29–1.10)	0.37 (0.17–0.79)
Multivariable adjusted model 1	1.00 (Ref.)	0.57 (0.29–1.12)	0.39 (0.18–0.86)
Multivariable adjusted model 2	1.00 (Ref.)	0.54 (0.27–1.08)	0.40 (0.18–0.89)
Multivariable adjusted model 3	1.00 (Ref.)	0.54 (0.27–1.09)	0.41 (0.19–0.92)
Multivariable adjusted model 4	1.00 (Ref.)	0.56 (0.28–1.12)	0.45 (0.20–1.01)

^§^ Age strata as underlying time variable in all the models; *N* = 13,421; Ref: reference category. Multivariable adjusted **model 1**: Additionally adjusted for sex, body mass index (continuous), total energy intake (continuous), physical activity (continuous), television watching (continuous), smoking (never, current or former), family history of stroke, and treatment with aspirin. Multivariable adjusted **model 2**: Additionally adjusted for the number of cardiovascular-related diseases at baseline, prevalent cancer, prevalent hypertension, prevalent diabetes, prevalent hypercholesterolemia, and prevalent hypertrygliceridemia. Multivariable adjusted **model 3**: Additionally adjusted for dietary fiber (fiber from foods other than fruits and vegetables) (continuous). Multivariable adjusted **model 4**: Additionally adjusted for the MDS without fruit and vegetable intake related items (low, medium, or high).

**Table 4:** Hazard Ratio (HR) and 95% Confidence Interval (CI) for the association of total vitamin C intake, adjusted for fiber intake using the residuals method with both cardiovascular disease (CVD) and cardiovascular mortality (CVM) for participants over 40 years old in the SUN cohort (follow-up 1999–2016).

Main Analyses ^§^	Tertiles of Vitamin C Intake		
	Q1 (*N* = 4474)	Q2 (*N* = 4474)	Q3 (*N* = 4473)
Incident CVD (person-time-1 at risk)	58 (49,706)	44 (49,080)	32 (48,186)
Multivariable adjusted ^§ ‡^	1.00 (Ref.)	0.86 (0.57–1.29)	0.74 (0.47–1.15)
Additionally adjusted for MDS	1.00 (Ref.)	0.86 (0.57–1.29)	0.74 (0.47–1.15)
Cardiovascular deaths (person-years at risk)	27 (49,879)	14 (49,247)	7 (48,368)
Multivariable adjusted ^§ ‡^	1.00 (Ref.)	0.52 (0.26–1.02)	0.30 (0.13–0.73)
Additionally adjusted for MDS	1.00 (Ref.)	0.52 (0.26–1.04)	0.30 (0.12–0.72)

MDS: Mediterranean Dietary Score without fruit and vegetable intake related items (low, medium, or high); ^§^ Age as underlying time variable in all the models; ^‡^ Adjusted for sex, body mass index (continuous), total energy intake (continuous), total fiber intake (continuous), physical activity (continuous), television watching (continuous), smoking (never, current or former), number of cardiovascular-related diseases at baseline, prevalent cancer, prevalent hypertension, prevalent diabetes, prevalent hypercholesterolemia, prevalent hypertriglyceridemia, family history of stroke, and treatment with aspirin. *N* = 13,421; Ref: reference category.

## Supplementary Materials

The following are available online at www.mdpi.com/2072-6643/9/9/954/s1, Figure S1: Hazard Ratio (HR) and 95% Confidence Interval (CI), for cardiovascular disease and cardiovascular mortality associated with vitamin C intake by vitamin C source in the SUN cohort (follow-up 1999–2016).
