# Supplementary figures and images for: Vitamin C Intake is Inversely Associated with Cardiovascular Mortality in a Cohort of Spanish Graduates: The SUN Project
Source: Nutrients. 2017 Aug 29;9(9):954. doi: 10.3390/nu9090954 (PMC5622714; doi:10.3390/nu9090954)

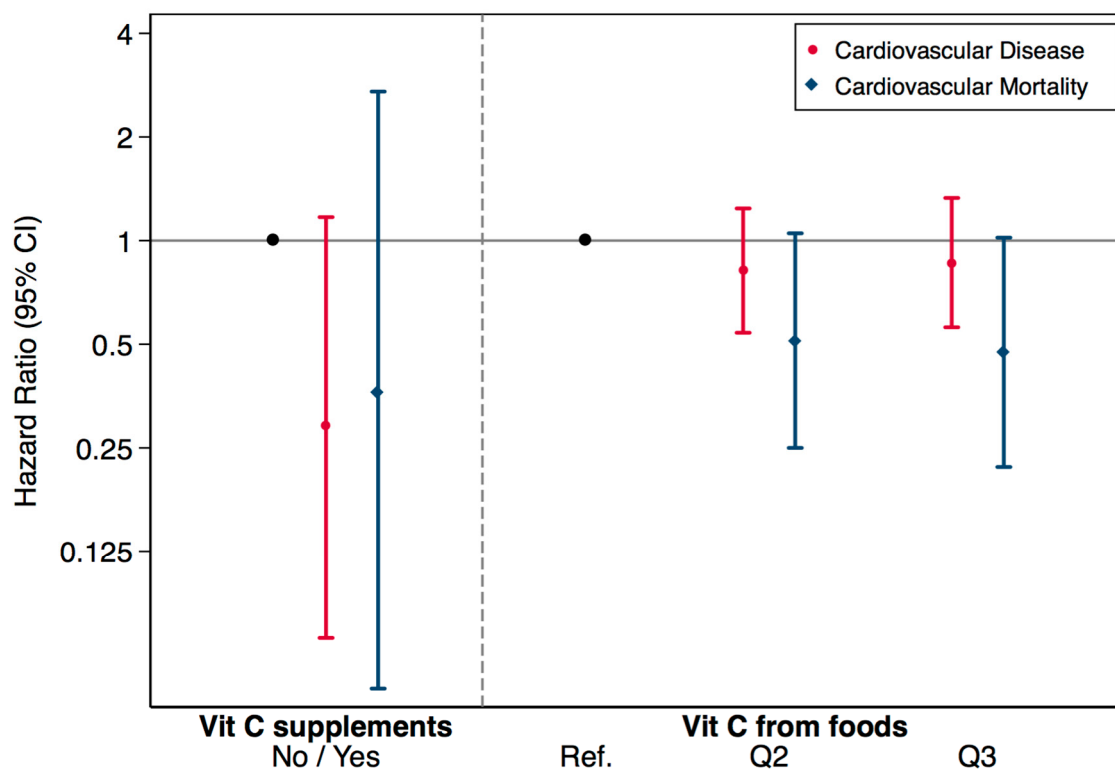

Supplement: Supplementary file 1 [file nutrients-09-00954-s001.pdf]
